# Early childhood exposure to environmental phenols and parabens, phthalates, organophosphate pesticides, and trace elements in association with attention deficit hyperactivity disorder (ADHD) symptoms in the CHARGE study

**DOI:** 10.1186/s12940-024-01065-3

**Published:** 2024-03-14

**Authors:** Jiwon Oh, Kyoungmi Kim, Kurunthachalam Kannan, Patrick J. Parsons, Agnieszka Mlodnicka, Rebecca J. Schmidt, Julie B. Schweitzer, Irva Hertz-Picciotto, Deborah H. Bennett

**Affiliations:** 1 Department of Public Health Sciences, University of California at Davis (UC Davis), Davis, CA USA; 2 UC Davis MIND (Medical Investigations of Neurodevelopmental Disorders) Institute, Sacramento, CA USA; 3 Division of Environmental Health Sciences, Wadsworth Center, New York State Department of Health, Albany, NY USA; 4 Department of Environmental Health Sciences, University at Albany, State University of New York, Albany, NY USA; 5 Department of Psychiatry and Behavioral Sciences, School of Medicine, University of California at Davis (UC Davis), Sacramento, CA USA

**Keywords:** ADHD, Environmental phenols, Parabens, Phthalates, Organophosphate pesticides, Trace elements, Mixtures

## Abstract

**Background:**

A growing body of literature investigated childhood exposure to environmental chemicals in association with attention-deficit/hyperactivity disorder (ADHD) symptoms, but limited studies considered urinary mixtures of multiple chemical classes. This study examined associations of concurrent exposure to non-persistent chemicals with ADHD symptoms in children diagnosed with autism spectrum disorder (ASD), developmental delay (DD), and typical development (TD).

**Methods:**

A total of 549 children aged 2–5 years from the Childhood Autism Risks from Genetics and Environment (CHARGE) case-control study were administered the Aberrant Behavior Checklist (ABC). This study focused on the ADHD/noncompliance subscale and its two subdomains (hyperactivity/impulsivity, inattention). Sixty-two chemicals from four classes (phenols/parabens, phthalates, organophosphate pesticides, trace elements) were quantified in child urine samples, and 43 chemicals detected in > 70% samples were used to investigate their associations with ADHD symptoms. Negative binomial regression was used for single-chemical analysis, and weighted quantile sum regression with repeated holdout validation was applied for mixture analysis for each chemical class and all chemicals. The mixture analyses were further stratified by diagnostic group.

**Results:**

A phthalate metabolite mixture was associated with higher ADHD/noncompliance scores (median count ratio [CR] = 1.10; 2.5th, 97.5th percentile: 1.00, 1.21), especially hyperactivity/impulsivity (median CR = 1.09; 2.5th, 97.5th percentile: 1.00, 1.25). The possible contributors to these mixture effects were di-2-ethylhexyl phthalate (DEHP) metabolites and mono-2-heptyl phthalate (MHPP). These associations were likely driven by children with ASD as these were observed among children with ASD, but not among TD or those with DD. Additionally, among children with ASD, a mixture of all chemicals was associated with ADHD/noncompliance and hyperactivity/impulsivity, and possible contributors were 3,4-dihydroxy benzoic acid, DEHP metabolites, MHPP, mono-n-butyl phthalate, and cadmium.

**Conclusions:**

Early childhood exposure to a phthalate mixture was associated with ADHD symptoms, particularly among children with ASD. While the diverse diagnostic profiles limited generalizability, our findings suggest a potential link between phthalate exposure and the comorbidity of ASD and ADHD.

**Supplementary Information:**

The online version contains supplementary material available at 10.1186/s12940-024-01065-3.

## Background

Attention-defici/hyperactivity disorder (ADHD) is a neurodevelopmental disorder, in which the individual manifests developmentally inappropriate levels of symptoms of inattention and/or hyperactivity/impulsivity [1]. Symptoms associated with the disorder occur on a continuum. ADHD is highly common, with the prevalence ranging from 5.9% [2] to 9.4% [3] and is twice as common in males as in females [2]. Because ADHD symptoms are increasingly diagnosed in the autism population, with the publication of the Diagnostic and Statistical Manual–5 (DSM-5) edition permitting the co-morbid diagnosis of ADHD to be given in autistic individuals [1], it is important to understand what factors might influence the presence of ADHD symptoms in autistic as well as non-autistic individuals. Estimates of ADHD symptoms in autism vary with older studies finding lower estimates, for example 2% [4], and more recent studies, as high as 78% [5]. The importance of studying autistic youth with significant ADHD symptoms is reinforced by findings from a recent study indicating 1.2% of children in the U.S. have both disorders [6]. Our group found that the rate of ADHD symptoms in children diagnosed with autism as well as with neurodevelopmental disorders who do not have autism is significantly higher than expected in the general population [7].

Despite the high heritability of ADHD, environmental factors, including chemical exposures, nutrient deficiencies, preterm birth, pregnancy complications, and extreme deprivation, are also associated with development of ADHD [8, 9]. While the prenatal period has been recognized as the most sensitive window of neurodevelopment, chemical exposure during the early postnatal period has also been a focus due to the continued postnatal development of the brain [10, 11]. An accumulating body of epidemiological literature suggests that prenatal as well as early-life exposures to environmental chemicals are associated with ADHD diagnosis or symptoms [12, 13].

Young children are exposed to mixtures of non-persistent environmental chemicals, including environmental phenols and parabens, phthalates, organophosphate (OP) pesticides, and trace elements [14]. Many of these chemicals have the potential to induce neurotoxicity and contribute to behavioral problems in laboratory animals [15–25], through mechanisms such as the disruption of thyroid hormone homeostasis [26–28], oxidative stress [29–31], or inhibition of the enzyme acetylcholinesterase in the brain [29, 30, 32]. However, epidemiological studies investigating childhood exposure to these chemicals, either as an individual compound or a mixture, in association with ADHD diagnosis or related behaviors have reported mixed results [33–50]. Additionally, there have been limited studies focusing on exposure to mixtures of environmental chemicals across multiple classes to address real-world exposures [51–53].

This study aimed to examine if concurrent exposure to each chemical as well as a mixture of these chemicals is associated with ADHD symptoms in early childhood in a cohort that includes children diagnosed with autism spectrum disorder (ASD) or developmental delay (DD) and those with typical development (TD).

## Methods

### Study population

Our study population consisted of a subset of children from the Childhood Autism Risks from Genetics and Environment (CHARGE) case-control study [54]. The CHARGE study primarily recruited children who received services for ASD or DD through the California Department of Developmental Services. General population controls were randomly selected from state birth files and frequency-matched to the sex, age, and residential catchment area of ASD cases. Given the male-to-female ASD prevalence ratio, the goal was to recruit more males (80%) than females (20%). Children were eligible for inclusion in the CHARGE study if they were 2 to 5 years old at enrollment, born in California, living with at least one biologic parent who speaks English or Spanish, and residing in the study catchment areas. Details on study design, subject recruitment, and data collection protocols are available elsewhere [54]. After being enrolled, children were administered a set of standardized assessments to confirm their diagnosis (Fig. S1). For example, children recruited as having ASD were clinically confirmed. Children recruited as having DD or controls were screened for ASD and evaluated for DD. Diagnostic tools and algorithms to classify children into ASD, DD, or TD groups are described elsewhere [54]. The study protocol received approval from the University of California (UC) Davis Institutional Review Boards and the State of California Committee for the Protection of Human Subjects. Before collecting data, participants provided written consent.

Among those who were enrolled between 2006 and 2017, a total of 549 children who provided a sufficient volume (≥ 16 mL) of urine and were assessed for ADHD behaviors were included in this study (Fig. S1). These children were classified as either ASD (*n* = 225), DD (*n* = 88), or TD (*n* = 236).

### Assessment of ADHD symptoms

Children at 2 to 5 years old were assessed for ADHD symptoms using the Aberrant Behavior Checklist (ABC) at the UC Davis Medical Investigations of Neurodevelopmental Disorders (MIND) Institute. The ABC was selected to assess behavioral symptoms because a substantial portion (57%) of the participants have intellectual disability. It was developed for children with neurodevelopmental concerns, particularly to assess the response to interventions [55]. The ABC has demonstrated moderate to high convergent validity with other commonly used scales, mostly in children with neurodevelopmental disorders [56, 57]. Furthermore, it showed good validity in children with ASD and TD [56] and in toddlers with neurodevelopmental disorders [58]. The ABC consists of 58 items, each of which is scored from 0 (not at all a problem) to 3 (the problem is severe in degree) with higher scores indicating greater problems [55]. The current study used the ADHD/noncompliance subscale of the ABC as the subscale items align most closely with those on the DSM-5 Text Revision (DSM-5-TR) [1] for ADHD, as opposed to the other subscales (Irritability, lethargy, stereotypy, and inappropriate speech). The ADHD/noncompliance subscale is composed of 16 items with a score range of 0–48 to assess ADHD symptoms. The ADHD/noncompliance subscale was further separated into two subdomains to explore the ADHD symptoms by subtypes: hyperactivity/impulsivity (10 items with a score range of 0–30) and inattention (3 items with a score range of 0–9) [7]. Items related to defiance and oppositionality were excluded, as according to both the current and most recent previous DSM-5-TR [1], Oppositional and Defiant Disorder is considered a separate disorder. When considering other commonly used behavioral instruments, such as Child Behavior Checklist [59] or Conners’ Parent Rating Scale [60], those items are assessed on separate scales. The list of items that belong to the ADHD/noncompliance subscale and two subdomains is shown in Table S1.

### Urinary chemical quantification

Child spot urine samples were collected at the study visit when the child was 2 to 5 years of age. The urine samples were immediately stored at − 20 °C, and aliquots were shipped on dry ice to the New York State Department of Health’s Wadsworth Center’s Child Health Exposure Analysis Resource (CHEAR) Targeted Analysis Laboratory. A total of 62 trace organic chemicals were analyzed in urine within the CHEAR organic biomonitoring section at Wadsworth: 30 phenols/parabens, 20 phthalate metabolites, and 6 dialkylphosphate (DAP) metabolites of OP pesticides; 6 trace elements were measured in urine within the CHEAR inorganic biomonitoring section at Wadsworth. The chemical names and abbreviations of the 62 analytes are presented in Table S2. For analysis of phenols/parabens, urine samples were enzymatically deconjugated and extracted using liquid-liquid extraction and analyzed by high-performance liquid chromatography-tandem mass spectrometry (HPLC-MS/MS) [61–63]. For quantification of phthalate metabolites, urine samples were processed using enzymatic deconjugation followed by solid-phase extraction (SPE) prior to HPLC-MS/MS analysis [64, 65]. DAP metabolites were extracted from urine samples using SPE and analyzed by HPLC-MS/MS [66]. Trace elements in urine were analyzed within the CHEAR section of the Laboratory of Inorganic and Nuclear Chemistry at Wadsworth using Inductively Coupled Plasma Mass Spectrometry (ICP-MS) [67]. Detailed descriptions of the analytical method for each chemical class, including sample preparation, instrumental analysis, and mass spectrometric parameters, are available elsewhere [68].

Fifteen blinded duplicates were analyzed with study samples, along with multiple CHEAR reference materials, for quality assurance purposes. Median relative percentage differences of the valid duplicate samples, in which both were detected above the limit of detection (LOD), ranged from 5 to 46% for phenols/parabens, 5 to 38% for phthalate metabolites, 8 to 13% for OP pesticide metabolites, and 1 to 27% for trace elements (Table S3). The LODs ranged from 0.02 to 1 ng/mL for phenols/parabens, 0.01 to 5 ng/mL for phthalate metabolites, 0.02 to 0.1 ng/mL for pesticide metabolites, and 0.0007 to 0.45 ng/mL for trace elements (Table S3). Instrument software-generated values were used for urinary chemical concentrations below the LOD to reduce bias from replacing non-detected concentrations with a single value [69, 70].

### Statistical analysis

#### Descriptive analysis

Among 62 analytes quantified in child urine samples, 43 chemicals with detection frequencies over 70%, including 21 phenols/parabens, 12 phthalates, 5 pesticides, and 5 trace elements, were included in the statistical analyses. Several zero or negative values, occurring as a result of blank correction of instrument software-generated values, were replaced with a fixed value (i.e., 0.0001) to allow natural log (ln)-transformation [71]. The positive nonzero values were then specific gravity (SG)-corrected using the following equation: *C*_*sg*_ = *C* × [(*SG*_*median*_ – 1)/(*SG* – 1), where *C*_*sg*_ is the SG-corrected chemical concentration, *C* is the measured chemical concentration, *SG*_*median*_ is the median (1.022) of SG values in this study samples, and *SG* is the measured SG value [72, 73].

Spearman’s rank correlation coefficients among SG-corrected concentrations of 43 compounds were computed. Mono-2-(carboxymethyl) hexyl phthalate (MCMHP), mono-(2-ethyl-5-carboxypentyl) phthalate (MECPP), mono (2-ethyl-5-hydroxyhexyl) phthalate (MEHHP), and mono (2-ethyl-5-oxohexyl) phthalate (MEOHP), originating from the same parent compound, di-2-ethylhexyl phthalate (DEHP), showed strong correlations. Similarly, benzophenones and their metabolites, including 2,4-dihydroxybenzophenone (BP1), 2-hydroxy-4-methoxybenzophenone (BP3), 2,2′-dihydroxy-4-methoxybenzophenone (BP8), and 4-hydroxybenzophenone (OH4BP), also showed strong correlations due to the common exposure sources. Therefore, molar sums of DEHP metabolites and benzophenones were separately computed and used in the subsequent analysis rather than individual compounds.

#### Covariate selection

Potential confounders and risk factors for ADHD were identified *a priori* based on a directed acyclic graph (Fig. S2) [74]. ADHD/noncompliance subscale and two subdomain scores and SG-corrected chemical concentrations were compared by covariates using the Wilcox rank-sum or the Kruskal-Wallis test, and those variables that had associations with all three outcomes (*p* < 0.05) were selected as covariates. The final set of covariates includes: CHARGE case-control study frequency matching factors (child’s sex [female, male], age at assessment [in years; centered to the mean], and recruitment regional center), child’s birth year (2000–2004, 2005–2008, 2009–2013) and race/ethnicity (non-Hispanic White, non-Hispanic non-White, Hispanic) as an indicator of structural racism, maternal metabolic conditions (healthy weight/overweight and no metabolic conditions, obese or hypertensive disorder/gestational diabetes), parity (1, ≥ 2), highest education in household (high school/GED or less, some college credit, bachelor’s degree or higher) as an indicator of socioeconomic status, and diagnostic groups (ASD, DD, TD). Among the indicator variables of socioeconomic status, which were weak to moderately correlated with each other, parental education was selected, instead of mother’s age at delivery and homeownership, to avoid collinearity issues because it was most strongly associated with both exposures and outcomes.

#### Single-chemical analysis

Negative binomial regression models, adjusting for the covariates, were used to examine the associations of each chemical with the ADHD/noncompliance subscale and two subdomain scores to account for over-dispersed count outcomes. The SG-corrected concentrations were ln-transformed and standardized prior to the regression analyses. Count ratios (CRs) and 95% confidence intervals (CIs) were computed by exponentiating regression coefficients. The corresponding *p*-values were corrected for multiple comparisons using the false discovery rate (FDR) method per outcome and chemical class.

#### Mixture analysis

Repeated holdout validation for weighted quantile sum (WQS) regression for negative binomial outcomes was implemented to investigate the associations of each chemical class mixture with ABC scores [75]. For a WQS regression, the empirical weights, indicating the relative importance, of each chemical were estimated across 100 bootstrap samples in the randomly partitioned training set (40%). In the remaining test set (60%), the WQS index, representing the total body burden, was computed per each chemical class using the estimated weights [76, 77]. The WQS index was used in negative binomial regression models with adjustment of the covariates to examine its association with the outcomes. To obtain stable WQS estimates, the repeated holdout validation approach was used by randomly partitioning the dataset 100 times and performing the WQS regression on each set, generating 100 effect estimates and chemical weights and taking the median as the final estimate [75]. By iterating the partitioning process 100 times, this approach improves generalizability by mitigating the impact of sample-specific chemical weights and WQS index estimates and addressing the potential for unbalanced partitions and biased estimates from a single partition. Our focus was on the positive direction, as our hypothesis posited that the mixture index would be associated with higher ABC scores (i.e., greater behavioral problems). When a chemical class mixture showed significant associations in 95% of the repetitions (i.e., CR between the 2.5th and 97.5th percentiles [PCT] indicating either CR > 1 or CR < 1), its chemical weight distribution was presented. Based on the Busgang Criteria, chemicals that had 90 and 50% of the repetitions above their class threshold were defined as probable and possible contributors, respectively [68, 78]. For example, if a phthalate metabolite exceeded the class threshold (1/9 phthalate metabolites = 0.11) in over 50% of the repetitions, the metabolite was considered as a possible contributor.

Associations between total mixtures of all 43 analytes, and ABC scores were investigated using random subset WQS with repeated holdout validation, which iteratively selects random subsets of 7 chemicals (√43 ~ 7) and estimates weight parameters by combining results across multiple ensemble steps [75, 79].

#### Stratified/effect modification analysis

As children with ASD, followed by those with DD, showed more ADHD symptoms when compared to those with TD [7, 58], the mixture analyses were stratified by diagnostic group (i.e., ASD, DD, and TD). Furthermore, as previous studies reported sex-specific associations of phenols, phthalates, OP pesticides, and trace elements with child neurodevelopment [13, 80–83], child’s sex was evaluated as an effect modifier in the mixture models for ADHD/noncompliance. Sex-stratified interaction WQS regression models, with 100 repeated holdouts, were constructed by including the interaction term between sex and WQS index in addition to their main effects and covariates [78, 84]. These models allow for sex-specific effect estimates and chemical weights.

All analyses were performed with an open-source R software, version 4.1.0 (R Foundation for Statistical Computing, Vienna, Austria), including the “gWQS” package [85]. A statistical significance level was set at 0.05 for unadjusted *p*-values and 0.10 for FDR-corrected *p*-values.

## Results

### ABC scores by demographic characteristics

The majority of the study children were males (80.1%) and born non-preterm (87.6%), and approximately 49% of them were non-Hispanic white (Table 1). More than half of the children were born to mothers who were not obese in pre-pregnancy and did not have any metabolic conditions (63.8%) and were multiparous (56.1%). Most of the participating families had a highest education level of a bachelor’s degree or higher (56.6%) and owned a home (60.4%).

**Table 1:** Aberrant Behavior Checklist (ABC) ADHD/noncompliance subscale and two subdomain scores by characteristics of 549 CHARGE children

Characteristics ^a^	All children (*n* = 549)	Aberrant Behavior Checklist (ABC)					
		ADHD/noncompliance (*n* = 515)		Hyperactivity/impulsivity (*n* = 520)		Inattention (*n* = 547)	
	**Freq (%)**	**Median (IQR)**	***p*-value** ^**b**^	**Median (IQR)**	***p*-value** ^**b**^	**Median (IQR)**	***p*-value** ^**b**^
Sex			0.48		0.41		0.72
Male	440 (80.1)	7.0 (1.0, 18.8)		4.0 (1.0, 11.0)		2.0 (0.0, 3.8)	
Female	109 (19.9)	6.0 (1.0, 19.0)		3.0 (0.0, 10.0)		2.0 (0.0, 4.0)	
Child’s birth year			0.008		0.004		0.008
2000–2004	150 (27.3)	5.0 (1.0, 14.5)		3.0 (0.0, 10.0)		1.0 (0.0, 3.0)	
2005–2008	215 (39.2)	6.0 (1.0, 15.0)		3.0 (0.0, 10.0)		1.0 (0.0, 3.0)	
2009–2013	184 (33.5)	10.0 (3.0, 20.5)		6.0 (1.5, 13.5)		2.0 (0.0, 4.0)	
Preterm birth (< 37 weeks)			0.06		0.08		0.07
No	481 (89.4)	7.0 (1.0, 17.5)		4.0 (0.0, 10.0)		1.5 (0.0, 4.0)	
Yes	57 (10.6)	10.0 (3.3, 24.0)		6.0 (1.0, 14.3)		2.0 (1.0, 4.0)	
Child’s race/ethnicity			0.01		0.01		0.04
White (non-Hispanic)	271 (49.7)	5.0 (1.0, 16.0)		3.0 (0.0, 10.0)		1.0 (0.0, 3.0)	
Non-White (non-Hispanic)	115 (29.2)	10.0 (2.0, 24.5)		6.0 (1.0, 16.0)		2.0 (0.0, 4.0)	
Hispanic (any race)	159 (21.1)	7.5 (1.0, 15.0)		4.0 (1.0, 10.0)		2.0 (0.0, 3.0)	
Mother’s age at delivery			0.004		0.009		0.008
< 30 years	245 (44.6)	8.0 (1.0, 20.5)		4.0 (1.0, 13.0)		1.0 (0.0, 4.0)	
30–34 years	169 (30.8)	4.0 (0.5, 12.5)		2.0 (0.0, 8.0)		1.0 (0.0, 3.0)	
≥ 35 years	135 (24.6)	10.0 (3.0, 17.0)		5.5 (1.3, 10.0)		2.0 (0.0, 4.0)	
Maternal metabolic conditions			0.03		0.03		0.03
Healthy weight/overweight and no metabolic conditions	354 (66.3)	6.0 (1.0, 16.0)		3.0 (0.0, 10.0)		1.0 (0.0, 3.0)	
Obese or hypertensive disorder/gestational diabetes	180 (33.7)	8.0 (2.0, 21.8)		6.0 (1.0, 14.0)		2.0 (0.0, 4.0)	
Parity			< 0.001		< 0.001		< 0.001
1	228 (42.8)	9.0 (3.0, 22.0)		6.0 (1.0, 14.0)		2.0 (0.0, 4.0)	
≥ 2	305 (57.2)	5.0 (1.0, 14.0)		2.0 (0.0, 9.3)		1.0 (0.0, 3.0)	
Highest education in household			< 0.001		< 0.001		< 0.001
High school/GED or less	57 (10.4)	20.5 (8.5, 28.0)		12.0 (5.0, 19.0)		3.0 (2.0, 5.0)	
Some college credit	179 (32.6)	7.0 (1.0, 16.8)		4.0 (1.0, 10.0)		2.0 (0.0, 3.0)	
Bachelor’s degree or higher	313 (57.0)	5.0 (1.0, 15.0)		3.0 (0.0, 10.0)		1.0 (0.0, 3.0)	
Homeowner			0.008		0.007		0.002
No	166 (31.2)	9.0 (2.0, 22.3)		5.0 (1.0, 15.0)		2.0 (0.0, 4.0)	
Yes	366 (68.8)	6.0 (1.0, 15.8)		3.0 (0.0, 10.0)		1.0 (0.0, 3.0)	
Diagnostic groups			< 0.001		< 0.001		< 0.001
ASD	225 (41.0)	19.0 (10.0, 27.0)		11.0 (5.0, 18.0)		4.0 (2.0, 5.0)	
DD	236 (43.0)	8.5 (3.5, 19.0)		5.0 (2.0, 11.5)		2.0 (1.0, 4.0)	
TD	88 (16.0)	1.0 (0.0, 5.0)		1.0 (0.0, 3.0)		0.0 (0.0, 1.0)	
Recruitment regional center			0.43		0.23		0.43
Alta, Far Northern, Redwood Coast	268 (48.9)	7.0 (1.0, 16.0)		4.0 (0.0, 10.0)		1.0 (0.0, 3.0)	
North Bay, East Bay, San Andreas, Golden Gate	160 (29.2)	6.0 (1.0, 17.0)		3.0 (1.0, 10.0)		2.0 (0.0, 4.0)	
Valley Mt, Central Valley, Kern	120 (21.9)	10.0 (1.0, 21.0)		6.0 (1.0, 13.0)		2.0 (0.0, 4.0)	
	**Median (IQR)**	***r***_***sp***_^**c**^	***p*-value** ^**d**^	***r***_***sp***_^**c**^	***p*-value** ^**d**^	***r***_***sp***_^**d**^	***p*-value** ^**d**^
Child’s age at assessment	4.0 (3.4, 4.5)	0.12	0.005	0.13	0.004	0.11	0.01

*ADHD* Attention-deficit/hyperactivity disorder, *ASD* Autism spectrum disorder, *CHARGE* Childhood Autism Risks from Genetics and Environment, *DD* Developmental delay, *Freq* Frequency, *GED* General educational development, *IQR* Interquartile range, ***r***_***sp***_, Spearman correlation coefficient, *TD* Typical development

^a^Missing (*n*): preterm birth (11), child’s race/ethnicity (5), maternal metabolic condition (16), parity (19), homeowner (21)

^b^P-values from the Wilcox rank-sum test or the Kruskal-Wallis test

^c^Spearman’s rank correlation coefficients between child’s age and ABC scores or DEP concentrations

^d^*P*-values from the significance test of Spearman’s rank correlation coefficient

Median (interquartile range) for ABC scores in the whole study population were 7 (1, 19) for the ADHD/noncompliance subscale, 4 (1, 11) for the hyperactivity/impulsivity subdomain, and 2 (0, 4) for the inattention subdomain. The ABC scores differed by demographic characteristics (Table 1). Non-preterm children had lower ABC scores than children born pre-term, and non-Hispanic white and Hispanic children had lower scores compared to non-Hispanic, non-White (i.e., Asian, Black, and multi-racial) children. Children whose mothers were 30 to 34 years old at delivery had lower scores than those whose mothers were younger than 30 years or at or older than 35 years. Children born to mothers who were obese in pre-pregnancy or had hypertensive disorder or gestational diabetes had higher scores compared to those born to mothers who were not obese or did not have metabolic conditions. The first-born children had higher scores than the second- or later-born children. Children born to parents whose maximum education level was high school or less had higher scores than those born to parents with higher education. Children from families that owned a home had lower scores than those from families that did not. In terms of diagnostic groups, children with ASD had the highest, those with DD had the second highest, and those with TD had the lowest scores.

### Child urinary chemical concentrations

Detection frequency and distributions of SG-uncorrected concentrations of each chemical in child urine samples are presented in Table S3. Sixteen out of 30 phenols/parabens, 11 out of 20 phthalate metabolites, 5 out of 6 pesticide metabolites, and 4 out of 6 trace elements were detected in greater than 90% of the samples. Several chemicals within each class were significantly correlated with each other (Fig. S3). Specifically, benzophenones (BP1, BP3, and BP8) showed strong correlations, as did DEHP metabolites (MCMHP, MECPP, MEHHP, and MEOHP). Correlations were weak to moderate among other phthalate metabolites (Spearman’s rank correlation coefficients [***r***_***sp***_] = 0.22–0.69) and among pesticide metabolites (***r***_***sp***_ = 0.28–0.67), while they were moderate to strong among parabens (***r***_***sp***_ = 0.30–0.78). There were differences in urinary chemical concentrations across all demographic characteristics, particularly birth year for all chemical classes, child sex for phthalate metabolites, homeownership for pesticide metabolites, and diagnostic groups for trace elements (Fig. S4).

### Associations of individual chemical concentrations with ADHD/noncompliance subscale and two subdomain scores

**Fig. 1:**
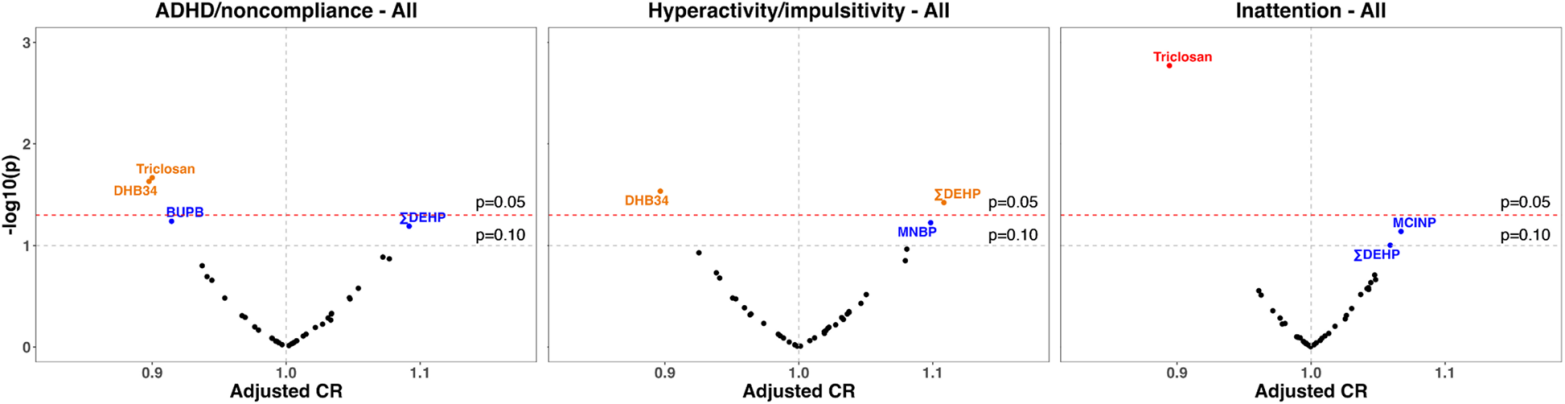
Volcano plots of covariate-adjusted CRs and unadjusted *p*-values of SG-corrected, ln-transformed, and standardized urinary chemical concentrations in association with ABC ADHD/noncompliance subscale and two subdomain scores among 549 CHARGE children. Red dots represent chemicals with an unadjusted *p* < 0.05 and an FDR-corrected *p* < 0.10, orange dots represent chemicals with an unadjusted *p* < 0.05, and blue dots represent chemicals with a 0.05 < unadjusted *p* < 0.10. Negative binomial regression models were adjusted for CHARGE case-control study frequency matching factors (child’s sex, age at assessment, and recruitment regional center), child’s birth year and race/ethnicity, parity, parental education, maternal metabolic conditions, and diagnosis. ABC, Aberrant Behavior Checklist; ADHD, attention-deficit/hyperactivity disorder; BUPB, butyl paraben; CHARGE, Childhood Autism Risks from Genetics and Environment; CR, count ratio; DEHP, di-2-ethylhexyl phthalate; DHB34, 3,4-dihydroxy benzoic acid; FDR, false discovery rate; MCINP, mono-carboxy isononyl phthalate; MNBP, mono-n-butyl phthalate; SG, specific gravity

There were several associations between individual urinary chemical concentrations and ABC scores, as shown in volcano plots (Fig. 1). Among all children, ∑DEHP was associated with higher scores of all three subscale/subdomains (CR = 1.09, 95% CI: 1.00, 1.20 for ADHD/noncompliance; CR = 1.11, 95% CI: 1.01, 1.22 for hyperactivity/impulsivity; CR = 1.06, 95% CI: 0.99, 1.13 for inattention) (Table S4). Two other phthalate metabolites were marginally associated with higher scores: mono-n-butyl phthalate (MNBP) with hyperactivity/impulsivity (CR = 1.10, 95% CI: 1.00, 1.21) and mono-carboxy isononyl phthalate (MCINP) with inattention (CR = 1.07, 95% CI: 0.99, 1.15). On the other hand, two phenols/parabens were associated with lower ABC scores: 3,4-dihydroxy benzoic acid (DHB34) with ADHD/noncompliance (CR = 0.90, 95% CI: 0.82, 0.99) and hyperactivity/impulsivity (CR = 0.90, 95% CI: 0.81, 0.99) and triclosan with ADHD/noncompliance (CR = 0.90, 95% CI: 0.82, 0.99) and inattention (CR = 0.89, 95% CI: 0.83, 0.96). However, after correcting for FDR, only the association between triclosan and inattention remained significant (Table S4). Pesticide metabolites and trace elements were not associated with ABC scores.

### Associations of chemical class and total mixtures with ADHD/noncompliance subscale and two subdomain scores

**Fig. 2:**
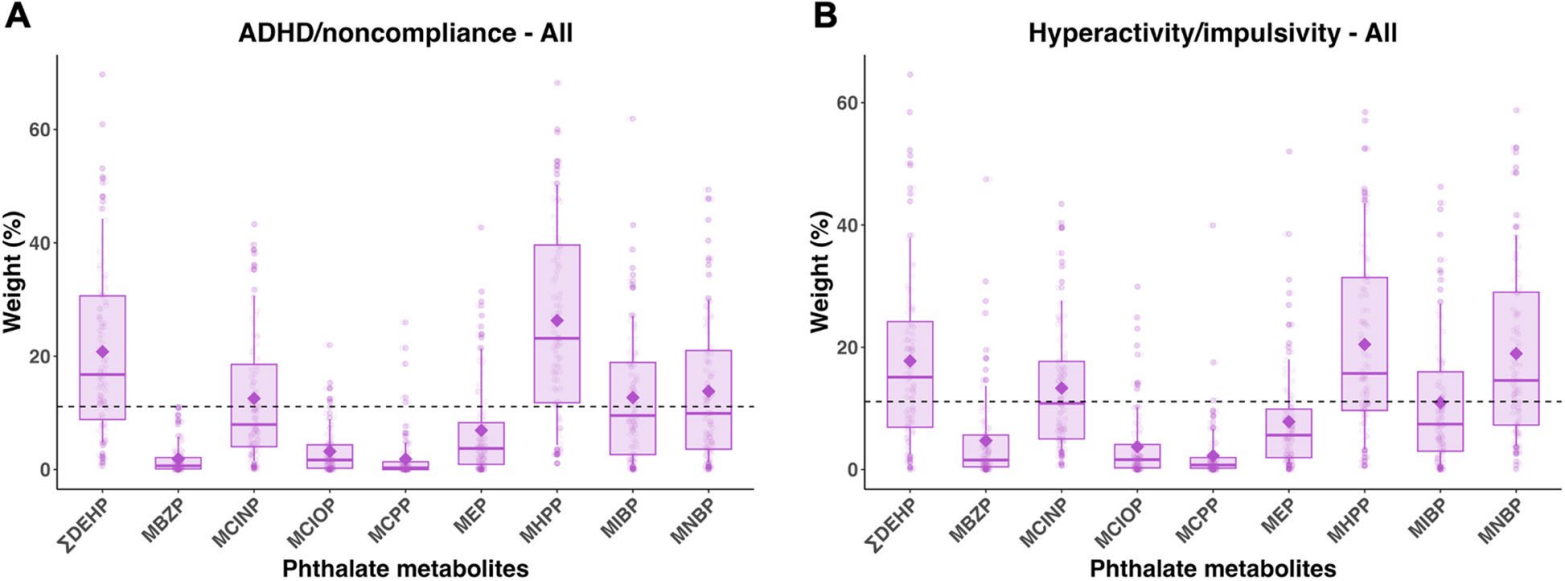
Estimated weight distributions of urinary phthalate metabolites from 100 repetitions of weighted quantile sum (WQS) regression for (A) ADHD/compliance subscale and (B) hyperactivity/impulsivity subdomain. Boxes indicate 25th, 50th, and 75th percentiles, diamonds indicate mean, and whiskers indicate 10th and 90th percentiles of weights. The dashed line indicates the threshold (1/# of chemicals in the mixture). ADHD, attention-deficit/hyperactivity disorder; DEHP, di-2-ethylhexyl phthalate; MBzP, mono-benzyl phthalate; MCINP, mono-carboxy isononyl phthalate; MCIOP, mono-carboxy isooctyl phthalate; MCPP, mono (3-carboxypropyl) phthalate; MEP, monoethyl phthalate; MHPP, mono-2-heptyl phthalate; MIBP, mono-isobutyl phthalate; MNBP, mono-n-butyl phthalate

Mixture analyses showed that the phthalate index was associated with higher scores of ADHD/noncompliance (median CR = 1.10, 2.5th and 97.5th PCT: 1.00, 1.21) and hyperactivity/impulsivity (median CR = 1.09, 2.5th and 97.5th PCT: 1.00, 1.25) among all children (Table 2). For both associations, ∑DEHP (17 and 15%, respectively) and mono-2-heptyl phthalate (MHPP) (23 and 16%, respectively) exceeded the class threshold (i.e., 1/9*100 = 11%) in over 50% of 100 repetitions and therefore were considered possible contributors based on the Busgang Criteria (Fig. 2). MNBP additionally contributed to the associations between the phthalate index and hyperactivity/impulsivity.

**Table 2:** Covariate-adjusted associations between mixtures and ABC ADHD/noncompliance subscale and two subdomain scores among all children and stratified by diagnostic group

Outcome	Mixture	All (*n* = 549)			ASD (*n* = 225)		
		Median CR^a^	2.5 PCT	97.5 PCT	Median CR ^b^	2.5 PCT	97.5 PCT
ADHD/noncompliance	Phenols/parabens	0.92	0.81	1.04	1.12	0.96	1.28
	Phthalate metabolites	**1.10**	**1.00**	**1.21**	**1.15**	**1.06**	**1.26**
	Pesticide metabolites	1.04	0.95	1.14	0.95	0.87	1.01
	Trace elements	0.97	0.87	1.10	1.04	0.89	1.15
	Total mixture	1.02	0.87	1.21	**1.15**	**1.01**	**1.29**
Hyperactivity/impulsivity	Phenols/parabens	0.92	0.75	1.07	1.07	0.92	1.25
	Phthalate metabolites	**1.09**	**1.00**	**1.25**	**1.22**	**1.07**	**1.37**
	Pesticide metabolites	1.01	0.91	1.11	0.98	0.88	1.04
	Trace elements	0.98	0.86	1.13	1.04	0.87	1.21
	Total mixture	1.03	0.84	1.18	**1.21**	**1.04**	**1.36**
Inattention	Phenols/parabens	0.97	0.85	1.09	1.11	0.97	1.25
	Phthalate metabolites	1.05	0.97	1.12	**1.10**	**1.02**	**1.20**
	Pesticide metabolites	0.98	0.93	1.07	0.99	0.91	1.08
	Trace elements	1.01	0.93	1.10	1.01	0.92	1.12
	Total mixture	0.98	0.85	1.08	1.11	0.96	1.26
		**TD (*n*** **= 236)** ^**c**^			**DD (*n*** **= 88)** ^**c**^		
		**Median CR** ^**b**^	**2.5 PCT**	**97.5 PCT**	**Median CR** ^**b**^	**2.5 PCT**	**97.5 PCT**
ADHD/noncompliance	Total mixture	1.01	0.69	1.51	0.75	0.47	1.18
Hyperactivity/impulsivity	Total mixture	1.05	0.61	1.40	0.75	0.48	1.40
Inattention	Total mixture	1.00	0.59	1.46	0.81	0.53	1.12

*ABC* Aberrant Behavior Checklist, *ADHD* Attention-deficit/hyperactivity disorder, *ASD* Autism spectrum disorder, *CHARGE* Childhood Autism Risks from Genetics and Environment, *CR* Count ratio, *DD* Developmental delay, *PCT* percentile, *TD* Typical development, *WQS* Weighted quantile sum

^a^WQS regression models were adjusted for CHARGE case-control study frequency matching factors (child’s sex, age at assessment, and recruitment regional center), child’s birth year and race/ethnicity, parity, parental education, maternal metabolic conditions, and diagnosis

^b^WQS regression models were adjusted for CHARGE case-control study frequency matching factors (child’s sex, age at assessment, and recruitment regional center), child’s birth year and race/ethnicity, parity, parental education, and maternal metabolic conditions

^c^Repeated holdout WQS regression models of each chemical class did not converge among children with TD or DD; therefore, the results were not presented

**Fig. 3:**
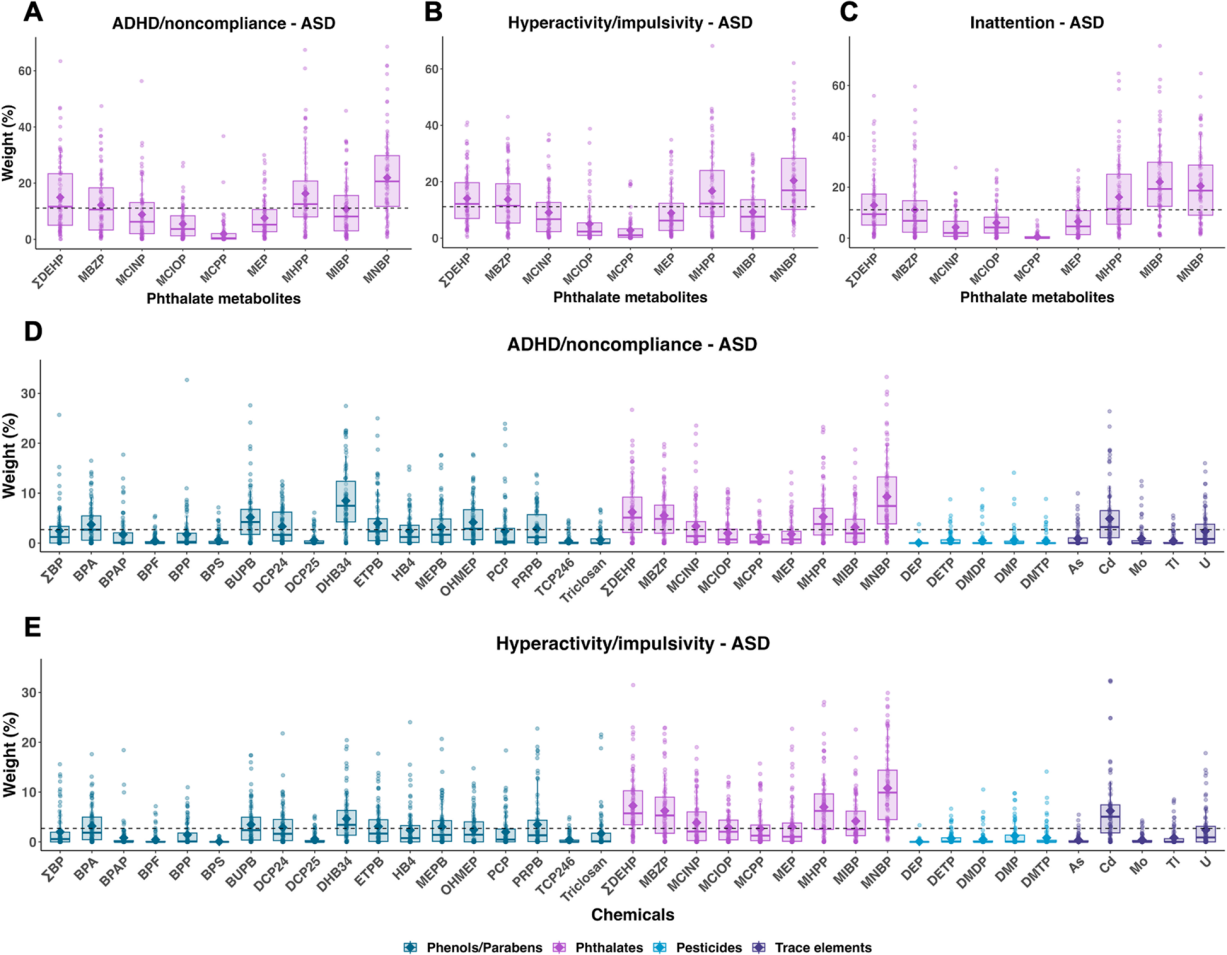
Estimated weight distributions of urinary chemicals from 100 repetitions of weighted quantile sum (WQS) regression, restricted to children with ASD. Phthalate metabolites in association with ADHD/noncompliance subscale, hyperactivity/impulsivity subdomain, and inattention subdomain are presented in (A), (B), and (C), respectively. Total chemicals in association with ADHD/noncompliance subscale and hyperactivity/impulsivity subdomain are presented in (D) and (E), respectively. Boxes indicate 25th, 50th, and 75th percentiles, diamonds indicate mean, and whiskers indicate 10th and 90th percentiles of weights. The dashed line indicates the threshold (1/# of chemicals in the mixture). Full chemical names are listed in Table S2. ADHD, attention-deficit/hyperactivity disorder; ASD, autism spectrum disorder

Stratified analysis by diagnostic group revealed several associations among children with ASD but not among children with DD or TD (Table 2). Among children with ASD, the phthalate index was also associated with higher scores of all three subscale/subdomains: ADHD/noncompliance (median CR = 1.15, 2.5th and 97.5th PCT: 1.06, 1.26), hyperactivity/impulsivity (median CR = 1.22, 2.5th and 97.5th PCT: 1.07, 1.37), and inattention (median CR = 1.10, 2.5th and 97.5th PCT: 1.02, 1.20). While ∑DEHP, mono-benzyl phthalate (MBZP), MHPP, and MNBP were common possible contributors for ADHD/noncompliance (12, 11, 13, and 21%, respectively) and hyperactivity/impulsivity (12, 12, 12, and 17%, respectively), MHPP, mono-isobutyl phthalate (MIBP), and MNBP were possible contributors for inattention (12, 19, and 19%, respectively) (Fig. 3). The total mixture index, of which DHB34, ∑DEHP, MBZP, MHPP, MNBP, and cadmium (Cd) were identified as possible contributors, was associated with higher scores of ADHD/noncompliance (median CR = 1.15, 2.5th and 97.5th PCT: 1.01, 1.29; median weight: 8, 5, 5, 4, 7, and 3%, respectively) and hyperactivity/impulsivity (median CR = 1.21, 2.5th and 97.5th PCT: 1.04, 1.36; median weight: 3, 6, 5, 6, 10, and 5%, respectively) (Table 2 and Fig. 3). The WQS regression models for each chemical class restricted to DD or TD did not converge (Table 2). Only the models for total mixtures converged, but none of them showed significant associations.

Sex-stratified interaction WQS regression models revealed no significant effect modification by child’s sex for associations between any mixture and ABC scores, as evaluated using the 2.5th and 97.5th PCTs of the interaction term between WQS index and child’s sex (Table S5). However, the phthalate index was associated with higher scores of ADHD/noncompliance among males only (median CR = 1.32, 2.5th and 97.5th PCT: 1.01, 2.70), with ∑DEHP and MHPP identified as possible contributors (Fig. S5a). On the other hand, the pesticide index was associated with higher scores of ADHD/noncompliance among females only (median CR = 1.28, 2.5th and 97.5th PCT: 1.03, 2.69), with diethylthiophosphate and dimethyldithiophosphate identified as possible contributors (Fig. S5b).

## Discussion

In the present study, concurrent measurement of environmental phenols and parabens, phthalates, OP pesticides, and trace elements in child urine samples were examined in association with ADHD symptoms, specifically the ADHD/noncompliance subscale and the hyperactivity/impulsivity and inattention subdomains, among 2- to 5-year-old children diagnosed with either ASD, DD, or TD. In the single-chemical analysis, DEHP metabolites were cross-sectionally associated with increased hyperactivity and impulsivity, while triclosan with decreased inattention (Table 3). In the mixture analysis using WQS regression, exposure to phthalate mixtures was associated with ADHD symptoms, especially hyperactivity and impulsivity, and the possible chemicals of concern were DEHP metabolites, MHPP, and MNBP.

**Table 3:** Summary table of associations between single chemical or mixtures and ADHD/noncompliance subscale and two subdomain scores among all children and children with ASD

Outcome	Chemical class	All (*n* = 549)		ASD (*n* = 225)
		Each chemical ^a^	Mixture ^b^	Mixture ^b^
ADHD/ noncompliance subscale	Phenols/ parabens	DHB34 (−) Triclosan (−)		
	Phthalate metabolites		∑DEHP (+) MHPP (+)	∑DEHP (+) MHPP (+) MNBP (+)
	Total mixture			DHB34 (+) BUPB (+) OHMEP (+) ∑DEHP (+) MBZP (+) MHPP (+) MNBP (+) Cd (+)
Hyperactivity/impulsivity subdomain	Phenols/ parabens	DHB34 (−)		
	Phthalate metabolites	∑DEHP (+)	∑DEHP (+) MHPP (+) MNBP (+)	∑DEHP (+) MHPP (+) MBZP (+) MNBP (+)
	Total mixture			DHB34 (+) ∑DEHP (+) MBZP (+) MHPP (+) MNBP (+) Cd (+)
Inattention subdomain	Phenols/ parabens	**Triclosan (−)**		
	Phthalate metabolites			MHPP (+) MIBP (+) MNBP (+)

Full chemical names are listed in Table S2

*ADHD* Attention-deficit/hyperactivity disorder, *ASD* Autism spectrum disorder, *CR* Count ratio, *FDR* False discovery rate

^a^Associations with significant associations are presented. Item in bold indicates significance even after FDR correction. (+) represents increased CR and (−) represents decreased CR

^b^Possible contributors of mixtures that have significant associations with outcomes are presented. (+) represents increased CR and (−) represents decreased CR

 These findings were likely driven by children with ASD, as the associations remained similar among children with ASD, but not among children with DD or TD. In addition, among children with ASD, a mixture of phthalate metabolites, possibly contributed by MIBP and MNBP, were associated with greater inattention. Further, mixtures of all chemicals, including phenols and parabens, phthalates, OP pesticides, and trace elements, were associated with ADHD symptoms, especially hyperactivity and impulsivity, and common possible contributors were DHB34, DEHP metabolites, MBZP, MHPP, MNBP, and Cd. These findings suggest that the early childhood exposure to several phthalates, parabens, and cadmium may be associated with the comorbidity of ASD and ADHD. One possible reason why we observed these associations among children with ASD only is higher and more variable ABC scores compared to those with DD or TD. However, as children with ASD are likely to have different dietary habits, behaviors, and usage of personal care products [86, 87] resulting in different exposure patterns to these non-persistent chemicals, potential reverse causality cannot be ruled out. Further studies on chemical exposures in relation to diets and behaviors in children with ASD can help address these questions.

Our findings on associations between childhood phthalate exposure, as an individual compound or a mixture, and greater ADHD symptoms in young children are generally in line with previous studies. One prospective study on childhood phthalate exposure in association with ADHD-related behaviors reported that MNBP and monoethyl phthalate (MEP) as well as phthalate metabolite mixtures, possibly contributed by MCINP, MEP, and MBZP, were associated with more externalizing problems, indicating more hyperactivity, aggression, and conduct problems in children aged 2–8 years [88]. Another study observed cross-sectional associations of greater ADHD traits with MBZP at 2 years [89]. Most of the other cross-sectional studies examining ADHD diagnosis or related behaviors in middle-childhood or adolescence reported adverse associations with DEHP metabolites [90–94] and di-n-butyl phthalate metabolites [33, 92, 93, 95–97], while a few additional prospective studies did not find convincing associations [98–100]. Young children not only have different exposure patterns to phthalates from their mothers, as indicated by weak correlations of phthalate metabolite concentrations in young children with those in their mothers’ prenatal or postnatal urine samples [101, 102], but also higher body burden [103, 104]. Therefore, the accumulating epidemiological evidence warrants further longitudinal investigations on early childhood exposure to phthalates and ADHD-related behaviors to establish causality, particularly in prospective study settings.

Underlying mechanisms of phthalates’ effects on ADHD remain unclear. ADHD is associated with alterations in the dopamine system and associated brain regions, such as the striatum, and potentially, the midbrain [105–109]. Toxicological studies reported that rats or mice neonatally exposed to DEHP or dicyclohexyl phthalate had impaired tyrosine hydroxylase immunoreactivity within midbrain dopaminergic nuclei [18, 110]. Neonatal exposure of rats to DEHP or dibutyl phthalate expressed hyperactivity, concomitantly with alterations in gene expression in the midbrain and striatum [19, 22, 23]. In addition to the effect of phthalates on subcortical structures, cortical thickness is modestly thinner in children with ADHD and delayed in maturation in comparison to control participants [111, 112]. Among children with ADHD, DEHP metabolite concentrations were negatively correlated with cortical thickness in the right middle and superior temporal gyri, suggesting a possible role of DEHP in impaired brain structures [93].

Significant associations of a phthalate metabolite mixture with ADHD symptoms were observed among males only, with no evident effect modification by sex. However, given that this study population includes four times more males than females, thus potentially underpowered for detecting associations in females, these findings should be interpreted with caution. Phthalates are reported to interfere with thyroid functions, which are essential for normal brain development, in a sexually dimorphic manner [26, 28], and early thyroid hormone disruption may contribute to the development of ADHD [113]. Still, regarding phthalate exposure and ADHD-related behaviors, there is inconsistent evidence on effect modification by sex [81, 88, 90, 91, 96, 97] or mediation by thyroid hormone [114]; therefore, these should be explored in future studies.

There are a limited number of studies examining associations of prenatal or childhood exposure to mixtures of multiple classes of urinary chemicals with ADHD diagnosis or related behaviors. Guilbert et al., who quantified phthalate/plasticizer metabolites and phenols/parabens in 416 prenatal maternal urine samples, observed that a chemical mixture, primarily weighted for BP3, triclosan, methyl paraben (MEPB), ethyl paraben (ETPB), and several phthalate metabolites (diisononyl phthalate metabolites, di (isononyl)cyclohexane-1,2-dicarboxylate metabolites, MBZP, MEP), was associated with more externalizing behaviors in 2-year-old French children [115]. Van den Dries et al. reported null associations of prenatal exposure to mixtures of phthalates, BPA, and OP pesticides with attention problems in 782 Dutch children aged 6 years [116]. Maitre et al. that measured pre- and postnatal environmental exposures from outdoor, indoor, chemical, lifestyle and social domains in 1287 European mother-child pairs observed associations of prenatal exposure to an OP pesticide metabolite, dimethyl phosphate (DMP), with more externalizing symptoms at 6–11 years of age, while those of childhood DMP exposure with less ADHD symptoms [51]. Waits et al. examined concurrent exposure to phthalates, OP pesticides, and nonylphenol in relation to 76 ADHD diagnoses versus 98 controls in Taiwanese children aged 4–15 years. They observed associations of a chemical mixture, primarily contributed by two OP pesticide metabolites (DMP, diethyl phosphate [DEP]) and two phthalate metabolites (MEP, MBZP), with increased odds of ADHD [53]. Many of these chemicals, to which the general population is simultaneously exposed, have endocrine disrupting potentials and share common mechanisms, including disruption of thyroid and neurotransmitter functions [21, 26, 117, 118], and concentrations of these chemicals frequently measured in the urine are correlated within and across class [53, 68, 115, 116, 119, 120]. Therefore, mixture analyses using multiple chemical classes helps with understanding of mixture effects of environmental chemicals on child neurobehaviors.

This study was strengthened by quantification of 62 chemicals from four chemical groups in urine samples of young children. WQS was employed to examine associations of chemical mixtures with ADHD-related behaviors, allowing for modeling multiple chemical exposures, which were correlated with each other, and minimizing the multiple comparisons problem. However, several limitations should be noted. First, due to the cross-sectional design, our results do not represent causal effects of childhood chemical exposures on ADHD symptoms. Second, this study also relied on concentrations of non-persistent chemicals measured in a spot urine sample, which reflect recent exposure. In young children, several phenols, phthalate metabolites, OP pesticides, and trace elements showed moderate reproducibility over short-term periods but reduced reproducibility over longer time frames [121–127]. Third, as this study used child urine samples as an exposure matrix, instead of whole blood samples, several other trace elements, especially known neurotoxicants, were not able to be included as analytes. Fourth, though an array of sociodemographic variables were considered as covariates, there is potential residual confounding by unmeasured factors related to diet, lifestyle, or parental ADHD symptoms. Fifth, our results cannot be generalized to general population because approximately 57% of our study population included children with ASD or DD, who showed more ADHD symptoms than those with TD. Still, the diverse diagnostic profile enabled us to examine childhood exposure to chemical mixtures in association with the comorbidity of ASD and ADHD, while exploring their association with ADHD behaviors in typically developing children. As distinct exposure mixture patterns may have differential effects on children based on their susceptibility, further investigations into ADHD symptoms among children with neurodevelopmental disorders are warranted.

## Conclusions

In the CHARGE population, comprised of 2- to 5-year-old children diagnosed with ASD, DD, and TD, concurrent exposure to a phthalate mixture, highly weighted for DEHP metabolites and MHPP, was associated with greater ADHD symptoms, possibly driven by children with ASD. Among children with ASD, a mixture of all chemicals were associated with ADHD symptoms, and possible chemicals of concern were one phenol (DHB34), several phthalate metabolites (DEHP metabolites, MBZP, MHPP, and MNBP), and a trace element (Cd). Because children with ASD not only have more pronounced ADHD behaviors but also show different exposure patterns to non-persistent chemicals due to different diet and behaviors, further attention to exposure of these children to possible neurotoxicants are warranted. Future investigation on exposure to mixtures of larger number of chemicals that share similar exposure sources could better address real-world exposures, in association with ADHD symptoms.

### Supplementary Information

**Supplementary Material 1.**

## Data Availability

Lab and epidemiological data are hosted at the Human Health Exposure Analysis Resources (HHEAR) Data Center Repository (https://hheardatacenter.mssm.edu/).
