# Supplementary material for: Early childhood exposure to environmental phenols and parabens, phthalates, organophosphate pesticides, and trace elements in association with attention deficit hyperactivity disorder (ADHD) symptoms in the CHARGE study
Source: Environ Health. 2024 Mar 14;23:27. doi: 10.1186/s12940-024-01065-3 (PMC10938747; doi:10.1186/s12940-024-01065-3)
Supplement: Supplementary file 1 — Supplementary Material 1. [file 12940_2024_1065_MOESM1_ESM.docx]

**Supplemental Material**

**Early childhood exposure to phenols/parabens, phthalates, organophosphate pesticides, and trace elements in association with attention deficit hyperactivity disorder (ADHD) symptoms in the CHARGE study**

Jiwon Oh, Kyoungmi Kim, Kurunthachalam Kannan, Patrick J. Parsons, Agnieszka Mlodnicka, Rebecca J. Schmidt, Julie B. Schweitzer, Irva Hertz-Picciotto, Deborah H. Bennett

**Table of Contents**

**Table S1.** List of items for Aberrant Behavior Checklist (ABC) ADHD/noncompliance and twos.

**Table S2.** Chemical names and abbreviations of 62 analytes.

**Table S3.** Limit of detection (LOD), percent detection above the LOD, and distribution of specific gravity (SG)-uncorrected concentrations (ng/mL) of phenols/parabens, phthalate metabolites, pesticide metabolites, and trace elements in urine samples of 549 CHARGE children.

**Table S4.** Covariate-adjusted associations between individual chemicals and ABC subscale/subdomain scores and *p*-values with and without FDR correction for multiple testing among 549 CHARGE children.

**Table S5.** Covariate-adjusted associations between mixtures and ADHD/noncompliance scores, stratified by child’s sex.

**Figure S1.** Flowchart depicting study design and study population.

**Figure S2.** Directed acyclic graph for associations of child urinary chemical concentrations with attention deficit hyperactivity disorder (ADHD) symptoms.

**Figure S3.** Heatmap of Spearman’s correlation coefficients among specific gravity (SG)-corrected concentrations of 43 compounds.

**Figure S4.** P-value matrix from the Wilcoxon rank-sum test or Kruskal-Wallis test comparing specific gravity (SG)-corrected chemical concentrations by characteristics of 549 CHARGE children.

**Figure S5.** Estimated weight distributions of urinary (A) phthalate metabolites and (B) pesticide metabolites from 100 repetitions of sex-stratified interaction weighted quantile sum (WQS) regression for ADHD/noncompliance.

**Table S1.** List of items for Aberrant Behavior Checklist (ABC) ADHD/noncompliance and twos subdomains.

| ADHD/noncompliance | Hyperactivity/  impulsivity | Inattention |
| --- | --- | --- |
| 1. Excessively active at home, school, work, or elsewhere | O |  |
| 7. Boisterous (inappropriately noisy and rough) | O |  |
| 13. Impulsive (acts without thinking) | O |  |
| 15. Restless, unable to sit still | O |  |
| 18. Disobedient; difficult to control |  |  |
| 21. Disturbs others | O |  |
| 24. Uncooperative |  |  |
| 31. Disrupts group activities | O |  |
| 38. Does not stay in seat (e.g., during lesson or training periods, meals, etc.) | O |  |
| 39. Will not sit still for any length of time | O |  |
| 48. Constantly jumps or runs around the room | O |  |
| 54. Tends to be excessively active | O |  |
| 28. Does not pay attention to instructions |  | O |
| 44. Easily distractible |  | O |
| 51. Pays no attention when spoken to |  | O |
| 56. Deliberately ignores directions |  |  |

Abbreviations: ADHD, attention-deficit/hyperactivity disorder.

Note: Item no. 18, 24, and 56 were not used in the generation of the two subdomains.

**Table S2.** Chemical names and abbreviations of 62 analytes.

| **Abbreviation** | **Chemical name** |
| --- | --- |
| **Phenols/Parabens** | |
| BP1 | 2,4-dihydroxybenzophenone |
| BP2 | 2,2′,4,4′-tetrahydroxybenzophenone |
| BP3 | 2-hydroxy-4-methoxybenzophenone |
| BP8 | 2,2′-dihydroxy-4-methoxybenzophenone |
| BPA | Bisphenol A; 2,2-bis(4-hydroxyphenyl)propane |
| BPAF | 4,4′-(hexafluoroisopropylidene)-diphenol |
| BPAP | 4,4′-(1-phenylethylidene)bisphenol |
| BPB | 2,2-bis(4-hydroxyphenyl)butane |
| BPF | Bisphenol F; 4,4′-dihydroxydiphenylmethane |
| BPP | 4,4′-(1,4-phenylenediisopropylidene)bisphenol |
| BPS | Bisphenol S; 4,4′-sulfonyldiphenol |
| BPZ | 4,4′-cyclo-hexylidenebisphenol |
| BUPB | Butyl paraben |
| BZPB | Benzyl paraben |
| DCP24 | 2,4-dichlorophenol |
| DCP25 | 2,5-dichlorophenol |
| DHB34 | 3,4-dihydroxy benzoic acid |
| ETPB | Ethyl paraben |
| HB4 | 4-hydroxybenzoic acid |
| HEPB | Heptyl paraben |
| MEPB | Methyl Paraben |
| OH4BP | 4-hydroxybenzophenone |
| OHETP | Ethyl-protocatechuic acid |
| OHMEP | Methyl-protocatechuic acid |
| PCP | Pentachlorophenol |
| PRPB | Propyl paraben |
| TCC | Triclocarban |
| TCP245 | 2,4,5-trichlorophenol |
| TCP246 | 2,4,6-trichlorophenol |
| Triclosan | Triclosan |
| **Phthalate metabolites** | |
| MBZP | Mono-benzyl phthalate |
| MCHP | Monocyclohexyl phthalate |
| MCHPP | Mono(7-carboxyheptyl)phthalate |
| MCINP | Mono-carboxy isononyl phthalate |
| MCIOP | Mono-carboxy isooctyl phthalate |
| MCMHP | Mono-2-(carboxymethyl) hexyl phthalate |
| MCPP | Mono (3-carboxypropyl) phthalate |
| MECPP | Mono-(2-ethyl-5-carboxypentyl) phthalate |
| MEHHP | Mono (2-ethyl-5-hydroxyhexyl) phthalate |
| MEOHP | Mono (2-ethyl-5-oxohexyl) phthalate |
| MEP | Monoethyl phthalate |
| MHPP | Mono-2-heptyl phthalate |
| MHXP | Mono-hexyl phthalate |
| MIBP | Mono-isobutyl phthalate |
| MINP | Mono-isononyl phthalate |
| MIPP | Mono-isopropyl phthalate |
| MMP | Mono-methyl phthalate |
| MNBP | Mono-n-butyl phthalate |
| MOP | Mono-n-octyl phthalate |
| MPEP | Mono-pentyl phthalate |
| **Pesticide metabolites** | |
| DEDP | Diethyldithiophosphate |
| DEP | Diethylphosphate |
| DETP | Diethylthiophosphate |
| DMDP | Dimethyldithiophosphate |
| DMP | Dimethylphosphate |
| DMTP | Dimethylthiophosphate |
| **Trace elements** | |
| As | Arsenic |
| Be | Beryllium |
| Cd | Cadmium |
| Mo | Molybdenum |
| Tl | Thallium |
| U | Uranium |

**Table S3.** Limit of detection (LOD), percent detection above the LOD, analysis results of 15 blinded duplicate samples, and distribution of specific gravity (SG)-uncorrected concentrations (ng/mL) of phenols/parabens, phthalate metabolites, pesticide metabolites, and trace elements in urine samples of 549 CHARGE children.

| **Chemical** | **LOD** | **% above LOD** | **# of valid pairs ^a^** | **RPD ^b^ median (%)** | **5^th^ Percentile** | **25^th^ Percentile** | **Median** | **75^th^ Percentile** | **95^th^ Percentile** |
| --- | --- | --- | --- | --- | --- | --- | --- | --- | --- |
| **Phenols/parabens** | | | | | | | | | |
| BP1 | 0.05 | 100 | 15 | 11 | 1.3 | 4.5 | 13.8 | 56.7 | 6838.0 |
| BP2 | 0.2 | 11.8 | 1 | 35 | <LOD | <LOD | <LOD | <LOD | 0.5 |
| BP3 | 0.5 | 100 | 15 | 14 | 4.7 | 15.3 | 49.5 | 198.0 | 1582.0 |
| BP8 | 0.02 | 82.0 | 11 | 21 | <LOD | 0.1 | 0.2 | 0.8 | 11.1 |
| BPA | 0.05 | 98.5 | 13 | 17 | 0.2 | 1.1 | 2.2 | 4.3 | 12.6 |
| BPAF | 0.05 | 0.7 | 0 | - | <LOD | <LOD | <LOD | <LOD | <LOD |
| BPAP | 0.1 | 89.4 | 14 | 46 | <LOD | 0.2 | 0.3 | 0.5 | 1.1 |
| BPB | 0.05 | 50.3 | 5 | 43 | <LOD | <LOD | 0.1 | 0.1 | 0.3 |
| BPF | 0.5 | 89.3 | 13 | 12 | <LOD | 1.2 | 3.0 | 7.2 | 21.1 |
| BPP | 0.01 | 76.1 | 14 | 44 | <LOD | 0.01 | 0.1 | 0.2 | 0.7 |
| BPS | 0.05 | 94.2 | 15 | 13 | <LOD | 0.2 | 0.4 | 0.9 | 3.8 |
| BPZ | 0.05 | 1.3 | 0 | - | <LOD | <LOD | <LOD | <LOD | <LOD |
| BUPB | 0.05 | 92.3 | 15 | 26 | <LOD | 0.1 | 0.4 | 1.0 | 12.6 |
| BZPB | 0.1 | 14.9 | 4 | 32 | <LOD | <LOD | <LOD | <LOD | 0.3 |
| DCP24 | 0.02 | 95.3 | 15 | 17 | 0.1 | 1.2 | 2.6 | 5.8 | 13.9 |
| DCP25 | 0.02 | 88.2 | 10 | 17 | <LOD | 1.5 | 3.4 | 6.5 | 78.2 |
| DHB34 | 0.2 | 100 | 15 | 8 | 32.1 | 83.8 | 149.0 | 266.0 | 513.6 |
| ETPB | 0.02 | 99.1 | 15 | 15 | 0.2 | 0.6 | 1.2 | 3.1 | 58.0 |
| HB4 | 1 | 100 | 15 | 5 | 393.2 | 976.0 | 1470.0 | 2390.0 | 6408.0 |
| HEPB | 0.1 | 0.4 | 0 | - | <LOD | <LOD | <LOD | <LOD | <LOD |
| MEPB | 0.05 | 99.8 | 15 | 8 | 3.4 | 12.5 | 40.4 | 206.0 | 2678.0 |
| OH4BP | 0.2 | 98.9 | 15 | 13 | 0.5 | 1.1 | 1.8 | 2.9 | 12.1 |
| OHETP | 0.1 | 64.7 | 10 | 9 | <LOD | <LOD | 0.3 | 1.0 | 4.5 |
| OHMEP | 0.1 | 95.8 | 12 | 11 | 0.2 | 1.5 | 4.4 | 15.1 | 113.8 |
| PCP | 0.05 | 96.0 | 12 | 21 | 0.1 | 0.5 | 1.0 | 1.9 | 4.9 |
| PRPB | 0.1 | 98.5 | 15 | 18 | 0.4 | 1.8 | 5.7 | 25.5 | 383.6 |
| TCC | 0.1 | 7.5 | 2 | 8 | <LOD | <LOD | <LOD | <LOD | 0.3 |
| TCP245 | 0.1 | 49.9 | 6 | 35 | <LOD | <LOD | <LOD | 0.2 | 0.6 |
| TCP246 | 0.02 | 99.8 | 15 | 6 | 0.4 | 0.9 | 1.5 | 2.5 | 7.0 |
| Triclosan | 0.1 | 96.9 | 14 | 8 | 0.3 | 2.6 | 7.4 | 26.1 | 214.2 |
| **Phthalate metabolites** | | | | | | | | | |
| MBZP | 0.02 | 99.5 | 14 | 5 | 3.9 | 13.4 | 30.4 | 70.2 | 283.6 |
| MCHP | 0.5 | 8.0 | 0 | - | <LOD | <LOD | <LOD | <LOD | 0.8 |
| MCHPP | 0.1 | 26.6 | 4 | 5 | <LOD | <LOD | <LOD | 0.1 | 13.6 |
| MCINP | 0.01 | 98.4 | 15 | 11 | 0.9 | 3.2 | 5.4 | 10.3 | 29.8 |
| MCIOP | 0.01 | 99.8 | 15 | 6 | 3.2 | 9.8 | 20.0 | 40.2 | 111.8 |
| MCMHP | 0.02 | 98.5 | 15 | 25 | 1.8 | 8.0 | 16.1 | 33.8 | 94.5 |
| MCPP | 0.05 | 99.8 | 15 | 7 | 0.9 | 2.8 | 5.5 | 10.3 | 24.9 |
| MECPP | 0.02 | 99.6 | 15 | 8 | 5.7 | 17.3 | 33.4 | 68.2 | 211.8 |
| MEHHP | 0.2 | 100 | 15 | 7 | 7.0 | 21.9 | 44.3 | 97.1 | 270.2 |
| MEOHP | 0.01 | 100 | 15 | 9 | 2.6 | 9.0 | 18.9 | 40.3 | 118.6 |
| MEP | 0.1 | 100 | 15 | 5 | 9.3 | 20.9 | 41.3 | 76.1 | 317.0 |
| MHPP | 0.5 | 73.6 | 8 | 38 | <LOD | <LOD | 1.3 | 3.0 | 15.2 |
| MHXP | 0.5 | 55.9 | 6 | 14 | <LOD | <LOD | 0.7 | 8.9 | 160.0 |
| MIBP | 0.01 | 100 | 15 | 9 | 3.3 | 10.6 | 19.2 | 33.5 | 82.3 |
| MINP | 0.01 | 0.0 | 0 | - | <LOD | <LOD | <LOD | <LOD | <LOD |
| MIPP | 0.5 | 2.4 | 0 | - | <LOD | <LOD | <LOD | <LOD | <LOD |
| MMP | 5 | 40.8 | 5 | 36 | <LOD | <LOD | <LOD | 7.9 | 28.1 |
| MNBP | 0.2 | 99.8 | 15 | 7 | 7.2 | 21.0 | 39.7 | 72.4 | 184.4 |
| MOP | 0.5 | 1.3 | 0 | - | <LOD | <LOD | <LOD | <LOD | <LOD |
| MPEP | 1 | 2.2 | 0 | - | <LOD | <LOD | <LOD | <LOD | <LOD |
| **Pesticide metabolites** | | | | | | | | | |
| DEDP | 0.05 | 3.8 | 0 | - | <LOD | <LOD | <LOD | <LOD | <LOD |
| DEP | 0.1 | 99.1 | 15 | 8 | 0.5 | 1.6 | 3.6 | 8.6 | 28.1 |
| DETP | 0.02 | 98.5 | 15 | 13 | 0.0 | 0.2 | 0.5 | 1.3 | 5.0 |
| DMDP | 0.02 | 91.4 | 13 | 8 | <LOD | 0.1 | 0.3 | 0.9 | 7.3 |
| DMP | 0.05 | 99.5 | 15 | 8 | 0.9 | 3.3 | 8.1 | 20.3 | 70.2 |
| DMTP | 0.05 | 95.3 | 15 | 8 | 0.1 | 1.0 | 3.3 | 11.2 | 49.6 |
| **Trace elements** | | | | | | | | | |
| As | 0.059 | 100 | 15 | 1 | 2.7 | 6.4 | 10.8 | 17.1 | 44.5 |
| Be | 0.022 | 6.9 | 1 | 27 | <LOD | <LOD | <LOD | <LOD | 0.024 |
| Cd | 0.006 | 73.2 | 11 | 21 | <LOD | <LOD | 0.02 | 0.05 | 0.1 |
| Mo | 0.45 | 100 | 15 | 1 | 23.9 | 57.9 | 95.1 | 148.0 | 293.6 |
| Tl | 0.005 | 100 | 15 | 3 | 0.1 | 0.1 | 0.2 | 0.3 | 0.5 |
| U | 0.0007 | 99.6 | 15 | 3 | 0.002 | 0.006 | 0.01 | 0.02 | 0.05 |

Abbreviations: Full chemical names are listed in Table S2. ADHD, attention-deficit/hyperactivity disorder; ASD, autism spectrum disorder; CHARGE, Childhood Autism Risks from Genetics and Environment.

^a^ Number of blinded duplicate pairs where both were ≥LOD.

^b^ Relative percent differences (RPD) = |sample result – repeat result|/(sample result + repeat result)*100.

**Table S4.** Covariate-adjusted associations between individual chemicals and ABC subscale/subdomain scores and *p*-values with and without FDR correction for multiple testing among 549 CHARGE children.

| **Chemical** | **ADHD/noncompliance** | | | **Hyperactivity/impulsivity** | | | **Inattention** | | |
| --- | --- | --- | --- | --- | --- | --- | --- | --- | --- |
|  | **CR (95% CI) ^a^** | **Unadj.**  ***p*-value** | **FDR-corrected *p*-value** | **CR (95% CI) ^a^** | **Unadj.**  ***p*-value** | **FDR-corrected *p*-value** | **CR (95% CI) ^a^** | **Unadj.**  ***p*-value** | **FDR-corrected *p*-value** |
| **Phenols/parabens** | | | | | | | | | |
| ∑BP | 0.99 (0.90, 1.09) | 0.818 | 0.968 | 0.99 (0.89, 1.09) | 0.776 | 0.917 | 1.03 (0.95, 1.10) | 0.488 | 0.993 |
| BPA | 1.01 (0.93, 1.10) | 0.780 | 0.968 | 1.00 (0.91, 1.10) | 0.980 | 0.980 | 1.00 (0.94, 1.07) | 0.993 | 0.993 |
| BPAP | 0.98 (0.89, 1.07) | 0.633 | 0.942 | 0.95 (0.86, 1.05) | 0.329 | 0.854 | 1.00 (0.93, 1.07) | 0.964 | 0.993 |
| BPF | 0.98 (0.88, 1.08) | 0.680 | 0.942 | 0.96 (0.86, 1.07) | 0.481 | 0.854 | 1.01 (0.93, 1.09) | 0.862 | 0.993 |
| BPP | 0.97 (0.88, 1.07) | 0.491 | 0.942 | 0.96 (0.87, 1.07) | 0.473 | 0.854 | 1.04 (0.97, 1.13) | 0.271 | 0.993 |
| BPS | 1.00 (0.91, 1.11) | 0.968 | 0.968 | 0.99 (0.89, 1.10) | 0.893 | 0.946 | 0.98 (0.90, 1.06) | 0.593 | 0.993 |
| BUPB | 0.91 (0.84, 1.00) | 0.058 | 0.345 | 0.93 (0.84, 1.02) | 0.118 | 0.854 | 0.97 (0.90, 1.05) | 0.439 | 0.993 |
| DCP24 | 1.02 (0.93, 1.12) | 0.642 | 0.942 | 1.04 (0.94, 1.14) | 0.473 | 0.854 | 1.00 (0.92, 1.08) | 0.926 | 0.993 |
| DCP25 | 1.03 (0.93, 1.15) | 0.542 | 0.942 | 1.02 (0.91, 1.14) | 0.734 | 0.917 | 0.99 (0.91, 1.07) | 0.793 | 0.993 |
| DHB34 | **0.90 (0.82, 0.99)** | **0.023** | 0.211 | **0.90 (0.81, 0.99)** | **0.029** | 0.527 | 1.00 (0.92, 1.08) | 0.935 | 0.993 |
| ETPB | 0.94 (0.87, 1.03) | 0.220 | 0.659 | 0.97 (0.89, 1.07) | 0.585 | 0.877 | 0.98 (0.91, 1.05) | 0.586 | 0.993 |
| HB4 | 0.94 (0.86, 1.02) | 0.158 | 0.659 | 0.94 (0.85, 1.03) | 0.185 | 0.854 | 0.96 (0.89, 1.03) | 0.278 | 0.993 |
| MEPB | 0.94 (0.86, 1.03) | 0.202 | 0.659 | 0.95 (0.87, 1.05) | 0.335 | 0.854 | 0.99 (0.92, 1.07) | 0.880 | 0.993 |
| OHMEP | 1.00 (0.90, 1.10) | 0.948 | 0.968 | 0.99 (0.89, 1.09) | 0.815 | 0.917 | 1.03 (0.95, 1.11) | 0.528 | 0.993 |
| PCP | 1.01 (0.92, 1.10) | 0.902 | 0.968 | 1.02 (0.93, 1.12) | 0.705 | 0.917 | 0.96 (0.90, 1.04) | 0.308 | 0.993 |
| PRPB | 0.95 (0.87, 1.04) | 0.329 | 0.846 | 0.96 (0.87, 1.05) | 0.410 | 0.854 | 1.03 (0.96, 1.11) | 0.417 | 0.993 |
| TCP246 | 1.03 (0.93, 1.14) | 0.518 | 0.942 | 1.03 (0.93, 1.14) | 0.522 | 0.854 | 0.99 (0.92, 1.08) | 0.898 | 0.993 |
| Triclosan | **0.90 (0.82, 0.99)** | **0.022** | 0.211 | 0.94 (0.85, 1.04) | 0.209 | 0.854 | **0.89 (0.83, 0.96)** | **0.002** | **0.030** |
| **Phthalate metabolites** | | | | | | | | | |
| ∑DEHP | 1.09 (1.00, 1.20) | 0.064 | 0.405 | **1.11 (1.01, 1.22)** | **0.038** | 0.268 | 1.06 (0.99, 1.13) | 0.099 | 0.446 |
| MBZP | 0.97 (0.89, 1.05) | 0.510 | 0.765 | 1.00 (0.91, 1.09) | 0.951 | 0.951 | 0.99 (0.92, 1.07) | 0.806 | 0.910 |
| MCINP | 1.07 (0.97, 1.17) | 0.130 | 0.405 | 1.08 (0.98, 1.19) | 0.108 | 0.317 | 1.07 (0.99, 1.15) | 0.073 | 0.446 |
| MCIOP | 1.01 (0.92, 1.10) | 0.867 | 0.930 | 1.03 (0.94, 1.13) | 0.512 | 0.718 | 1.00 (0.94, 1.07) | 0.910 | 0.910 |
| MCPP | 1.00 (0.92, 1.10) | 0.930 | 0.930 | 1.02 (0.93, 1.13) | 0.638 | 0.718 | 0.99 (0.93, 1.06) | 0.811 | 0.910 |
| MEP | 0.99 (0.90, 1.10) | 0.875 | 0.930 | 1.03 (0.93, 1.14) | 0.604 | 0.718 | 1.01 (0.94, 1.09) | 0.734 | 0.910 |
| MHPP | 1.08 (0.98, 1.18) | 0.135 | 0.405 | 1.08 (0.98, 1.19) | 0.141 | 0.317 | 1.04 (0.97, 1.12) | 0.260 | 0.468 |
| MIBP | 1.03 (0.94, 1.13) | 0.466 | 0.765 | 1.05 (0.96, 1.15) | 0.304 | 0.547 | 1.05 (0.98, 1.12) | 0.195 | 0.468 |
| MNBP | 1.05 (0.96, 1.14) | 0.264 | 0.593 | 1.10 (1.00, 1.22) | 0.059 | 0.268 | 1.04 (0.98, 1.12) | 0.232 | 0.468 |
| **Pesticide metabolites** | | | | | | | | | |
| DEP | 1.01 (0.92, 1.09) | 0.869 | 0.909 | 1.02 (0.93, 1.12) | 0.689 | 0.813 | 1.01 (0.94, 1.08) | 0.777 | 0.950 |
| DETP | 1.05 (0.96, 1.15) | 0.336 | 0.789 | 1.05 (0.95, 1.15) | 0.371 | 0.759 | 1.05 (0.97, 1.13) | 0.217 | 0.950 |
| DMDP | 0.99 (0.90, 1.09) | 0.909 | 0.909 | 1.01 (0.91, 1.12) | 0.813 | 0.813 | 0.98 (0.91, 1.05) | 0.519 | 0.950 |
| DMP | 1.03 (0.95, 1.12) | 0.473 | 0.789 | 1.04 (0.95, 1.13) | 0.448 | 0.759 | 1.00 (0.93, 1.07) | 0.914 | 0.950 |
| DMTP | 1.05 (0.95, 1.15) | 0.327 | 0.789 | 1.04 (0.94, 1.14) | 0.455 | 0.759 | 1.00 (0.93, 1.08) | 0.950 | 0.950 |
| **Trace elements** | | | | | | | | | |
| As | 1.00 (0.92, 1.10) | 0.919 | 0.919 | 1.00 (0.91, 1.10) | 0.980 | 0.980 | 1.04 (0.97, 1.12) | 0.265 | 0.758 |
| Cd | 0.99 (0.91, 1.09) | 0.880 | 0.919 | 1.01 (0.92, 1.11) | 0.866 | 0.980 | 1.01 (0.94, 1.08) | 0.819 | 0.819 |
| Mo | 1.01 (0.93, 1.11) | 0.747 | 0.919 | 1.02 (0.93, 1.12) | 0.655 | 0.980 | 1.04 (0.97, 1.11) | 0.303 | 0.758 |
| Tl | 0.99 (0.89, 1.09) | 0.822 | 0.919 | 0.98 (0.89, 1.09) | 0.748 | 0.980 | 1.02 (0.95, 1.09) | 0.625 | 0.819 |
| U | 1.03 (0.93, 1.14) | 0.596 | 0.919 | 1.03 (0.93, 1.15) | 0.535 | 0.980 | 0.99 (0.92, 1.07) | 0.795 | 0.819 |

Abbreviations: Full chemical names are listed in Table S2. ABC, Aberrant Behavior Checklist; ADHD, attention-deficit/hyperactivity disorder; CHARGE, Childhood Autism Risks from Genetics and Environment; CR, count ratio; FDR, false discovery rate.

**^a^** Negative binomial regression models were adjusted for CHARGE case-control study frequency matching factors (child’s sex, age at assessment, and recruitment regional center), child’s birth year and race/ethnicity, parity, parental education, maternal metabolic conditions, and diagnosis.

**Table S5.** Covariate-adjusted associations between mixtures and ADHD/noncompliance scores, stratified by child’s sex.

| **Outcome** | **Mixture** | **Male (*n* = 440)** | | | **Female (*n* = 109)** | | | **Interaction (WQS*Sex)** | | |
| --- | --- | --- | --- | --- | --- | --- | --- | --- | --- | --- |
|  |  | **Median CR ^a^** | **2.5**  **PCT** | **97.5 PCT** | **Median CR ^a^** | **2.5**  **PCT** | **97.5 PCT** | **Median β ^a^** | **2.5**  **PCT** | **97.5 PCT** |
| ADHD/  noncompliance | Phenols/Parabens | 0.93 | 0.55 | 2.01 | 0.83 | 0.51 | 1.50 | 0.01 | -0.67 | 0.95 |
|  | Phthalate metabolites | **1.32** | **1.01** | **2.70** | 1.06 | 0.74 | 1.91 | 0.22 | -0.51 | 1.08 |
|  | Pesticide metabolites | 1.06 | 0.76 | 1.90 | **1.28** | **1.03** | **2.69** | -0.24 | -1.00 | 0.47 |
|  | Trace elements | 0.90 | 0.63 | 2.33 | 1.06 | 0.64 | 1.48 | -0.18 | -0.68 | 0.95 |
|  | Total mixture | 1.13 | 0.79 | 2.47 | 1.19 | 0.70 | 4.04 | -0.03 | -1.34 | 0.83 |

Abbreviations: ADHD, attention-deficit/hyperactivity disorder; CHARGE, Childhood Autism Risks from Genetics and Environment; CR, count ratio; FDR, false discovery rate, WQS, weighted quantile sum.

^a^ Sex-stratified interaction WQS regression models were adjusted for CHARGE case-control study frequency matching factors (child’s sex, age at assessment, and recruitment regional center), child’s birth year and race/ethnicity, parity, parental education, maternal metabolic conditions, diagnosis, and interaction term between sex and WQS index.

**
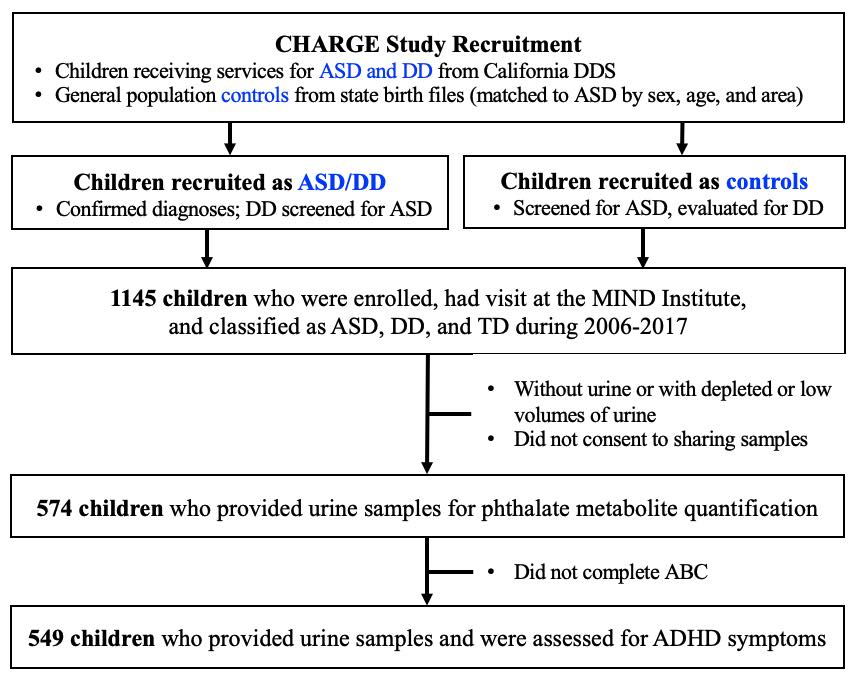
**

**Figure S1.** Flowchart depicting study design and study population. ABC, Aberrant Behavior Checklist; ADHD, attention-deficit/hyperactivity disorder; ASD, autism spectrum disorder; CHARGE, Childhood Autism Risks from Genetics and Environment; DD, developmental delay; DDS, department of developmental services; MIND, Medical Investigation of Neurodevelopmental Disorders; TD, typical development.

**
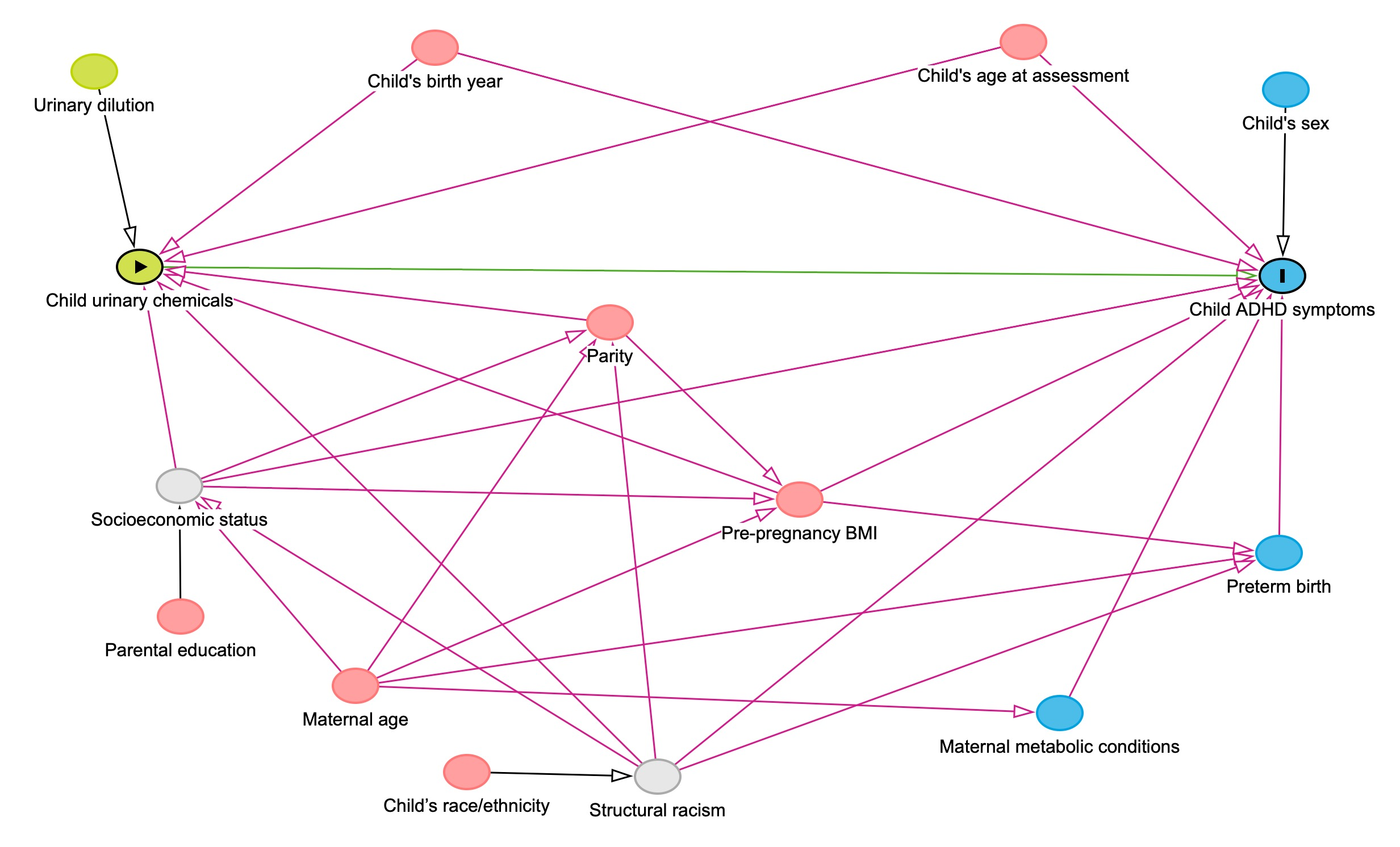
Figure S2.** Directed acyclic graph for associations of child urinary chemical concentrations with attention deficit hyperactivity disorder (ADHD) symptoms. Green circles indicate ancestors of the exposure, blue circles indicate ancestors of the outcome, pink circles indicate ancestors of both exposure and outcome, and grey circles indicate unobserved variables. BMI, body mass index.

**
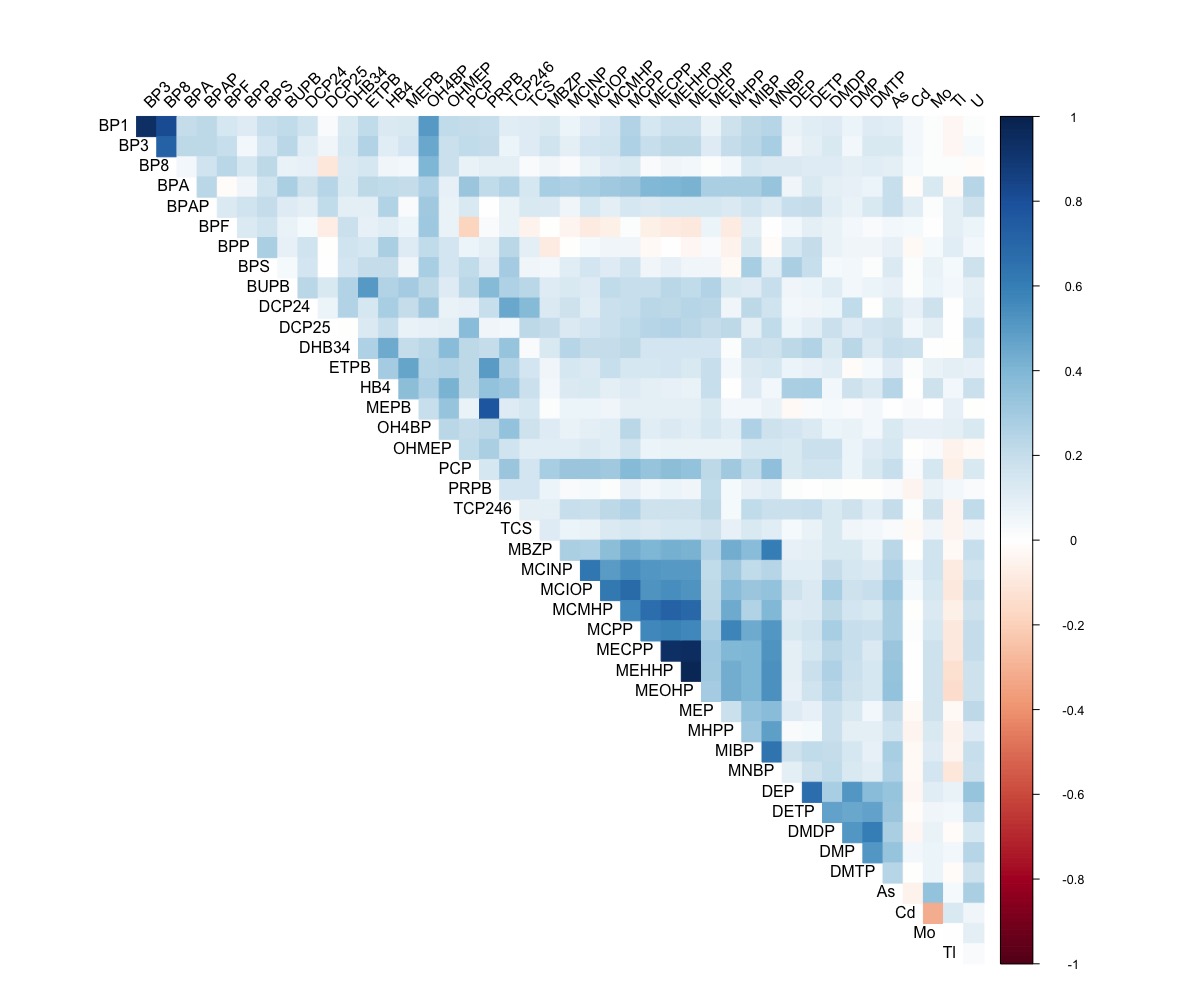
**

**Figure S3.** Heatmap of Spearman’s correlation coefficients among specific gravity (SG)-corrected concentrations of 43 compounds. Full chemical names are listed in Table S2.

**
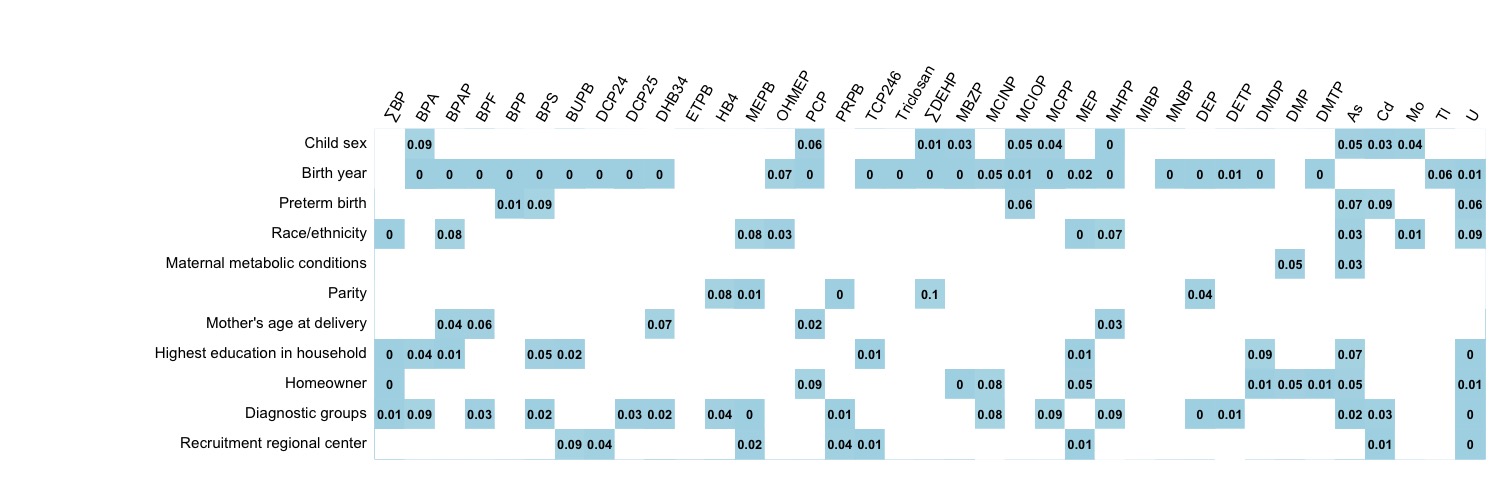
**

**Figure S4.** P-value matrix from the Wilcoxon rank-sum test or Kruskal-Wallis test comparing specific gravity (SG)-corrected chemical concentrations by characteristics of 549 CHARGE children. *P*-values less than 0.02 are presented. Full chemical names are listed in Table S2.

**
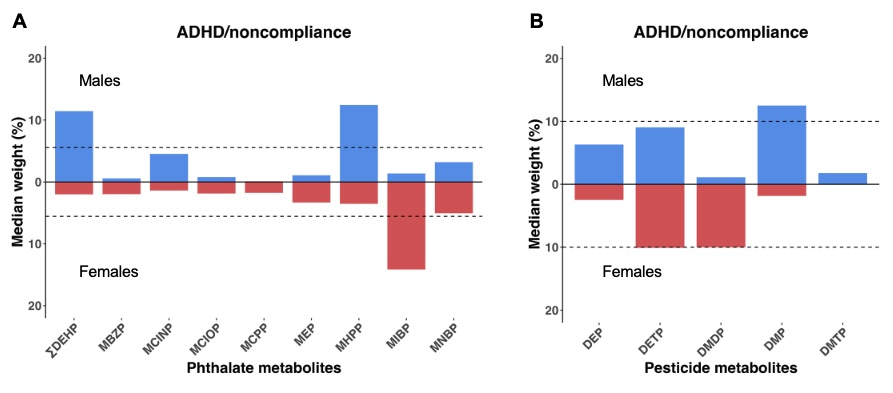
**

**Figure S5.** Estimated weight distributions of urinary (A) phthalate metabolites and (B) pesticide metabolites from 100 repetitions of sex-stratified interaction weighted quantile sum (WQS) regression for ADHD/noncompliance. Boxes indicate 50^th^ percentiles of weights. Dashed line indicates a threshold for sex [1/(# of chemicals in the mixture*2)]. Full chemical names are listed in Table S2. ADHD, attention-deficit/hyperactivity disorder.
